# Predictors of clinically significant prostate cancer in biopsy-naïve and prior negative biopsy men with a negative prostate MRI: improving MRI-based screening with a novel risk calculator

**DOI:** 10.1177/17562872221088536

**Published:** 2022-03-26

**Authors:** Luigi A.M.J.G. van Riel, Auke Jager, Dennie Meijer, Arnoud W. Postema, Ruth S. Smit, André N. Vis, Theo M. de Reijke, Harrie P. Beerlage, Jorg R. Oddens

**Affiliations:** Department of Urology, Prostate Cancer Network in the Netherlands, Amsterdam University Medical Centers, University of Amsterdam, Meibergdreef 9, 1105 AZ Amsterdam, The Netherlands; Department of Urology, Prostate Cancer Network in the Netherlands, Amsterdam University Medical Centers, University of Amsterdam, Amsterdam, The Netherlands; Department of Urology, Prostate Cancer Network in the Netherlands, Amsterdam University Medical Centers, University of Amsterdam, Amsterdam, The Netherlands; Department of Urology, Prostate Cancer Network in the Netherlands, Amsterdam University Medical Centers, University of Amsterdam, Amsterdam, The Netherlands; Department of Radiology, Amsterdam University Medical Centers, VU University, Amsterdam, The Netherlands; Department of Urology, Prostate Cancer Network in the Netherlands, Amsterdam University Medical Centers, University of Amsterdam, Amsterdam, The Netherlands; Department of Urology, Prostate Cancer Network in the Netherlands, Amsterdam University Medical Centers, University of Amsterdam, Amsterdam, The Netherlands; Department of Urology, Prostate Cancer Network in the Netherlands, Amsterdam University Medical Centers, University of Amsterdam, Amsterdam, The Netherlands; Department of Urology, Prostate Cancer Network in the Netherlands, Amsterdam University Medical Centers, University of Amsterdam, Amsterdam, The Netherlands

**Keywords:** biopsy, magnetic resonance imaging, prostatic neoplasms

## Abstract

**Purpose:**

A pre-biopsy decision aid is needed to counsel men with a clinical suspicion for clinically significant prostate cancer (csPCa), despite normal prostate magnetic resonance imaging (MRI).

**Methods:**

A risk calculator (RC) for csPCa (International Society of Urological Pathology grade group (ISUP) ⩾ 2) presence in men with a negative-MRI (Prostate Imaging–Reporting and Data System (PI-RADS) ⩽ 2) was developed, and its performance was compared with RCs of the European Randomized Study of Screening for Prostate Cancer (ERSPC), Prostate Biopsy Collaborative Group (PBCG), and Prospective Loyola University mpMRI (PLUM). All biopsy-naïve and prior negative biopsy men with a negative-MRI followed by systematic prostate biopsy were included from October 2015 to September 2021. The RC was developed using multivariable logistic regression with the following parameters: age (years), family history of PCa (first- or second-degree family member), ancestry (African Caribbean/other), digital rectal exam (benign/malignant), MRI field strength (1.5/3.0 Tesla), prior negative biopsy status, and prostate-specific antigen (PSA) density (ng/ml/cc). Performance of RCs was compared using receiver operating characteristic (ROC) curve analysis.

**Results:**

A total of 232 men were included for analysis, of which 18.1% had csPCa. Parameters associated with csPCa were family history of PCa (*p* < 0.0001), African Caribbean ancestry (*p* = 0.005), PSA density (*p* = 0.002), prior negative biopsy (*p* = 0.06), and age at biopsy (*p* = 0.157). The area under the curve (AUC) of the developed RC was 0.76 (95% CI 0.68–0.85). This was significantly better than the RCs of the ERSPC (AUC: 0.59; *p* = 0.001) and PBCG (AUC: 0.60; *p* = 0.002), yet similar to PLUM (AUC: 0.69; *p* = 0.09).

**Conclusion:**

The developed RC (Prostate Biopsy Cohort Amsterdam (‘PROBA’ RC), integrated predictors for csPCa at prostate biopsy in negative-MRI men and outperformed other widely used RCs. These findings require external validation before introduction in daily practice.

## Introduction

Diagnostic prostate magnetic resonance imaging (MRI) is well established as a diagnostic modality for men with a clinical suspicion for prostate cancer (PCa). Multiple (randomized) trials showed that performing a pre-biopsy prostate MRI increased the detection of clinically significant prostate cancer (csPCa).^1–4^ Consequently, current international guidelines strongly recommend performing prostate MRI before biopsy.^5^ Following a positive MRI (i.e. Prostate Imaging–Reporting and Data System (PI-RADS) classification of ⩾ 3), there is a clear indication for MRI-targeted biopsy that can be complemented with systematic biopsy (SBx).^6^ However, following a negative-MRI (i.e. PI-RADS ⩽ 2), there is no clear consensus on whether prostate biopsy should be performed.

Omitting SBx following a negative-MRI has the advantage of reducing diagnosis of insignificant prostate cancer (iPCa) and related overtreatment and avoiding prostate biopsy–related patient burden and morbidity.^6^ It also carries a risk of missing csPCa and related treatment delay, possibly leading to disease progression.^6^ To assist in clinical decision making, multiple risk calculators (RCs) have been developed that are able to predict csPCa presence. RCs are based on data of large cohorts and use a combination of patient-specific characteristics (e.g. age, digital rectal examination (DRE), prostate volume, prostate-specific antigen (PSA) levels, previous prostate biopsy results, prostate MRI results) to estimate the risk of having PCa at biopsy.^7^ RCs show significantly better predictive accuracy compared with individual variables, such as PSA alone. However, they suffer from limited generalizability and poor calibration with validation studies, often overestimating the risk for PCa.^7,8^ Specifically in men with negative-MRI, more data are needed to address the question in which of these men SBx can be safely omitted.^9^

Considering the aging population and shifting views on early detection, this topic will become more relevant. In the latest statement of the US Preventive Services Task Force, screening based on PSA for men aged 55–69 years is no longer discouraged.^10^ More screening will inevitably lead to an increased number of (negative) prostate MRIs, highlighting the need for reliable predictive factors for men at risk of significant disease, despite a negative-MRI.

Therefore, the aim of this study was to develop a RC for the presence of csPCa at SBx in men with a negative-MRI using a wide set of clinical parameters, and second, to compare the performance of the RC with that of widely used RCs.

## Patients and methods

In both participating centers, performing a pre-biopsy MRI was regularly performed for men with a clinical suspicion of PCa. All biopsy patients were prospectively registered from October 2015 and September 2021. Biopsy naïve or prior negative men were included in this subgroup analysis if their prostate MRI was negative (PI-RADS 1-2) and performed within 6 months prior to prostate biopsy. Men with prior PCa treatment or prior positive prostate biopsies were excluded. The study was approved by the Institutional Review Board of the participating centers (reference number: W21_534). The Ethical Committee confirmed that The Medical Research Involving Human Subjects Act (in Dutch: WMO) does not apply for this registry. As the current study exclusively re-uses care data for the purpose of research, written patient consent was not required.

### Clinical parameters

The following clinical parameters were used: age (years), family history of PCa (first- or second-degree family member), ancestry (African Caribbean/other), DRE (benign/malignant), MRI field strength (1.5/3.0 Tesla), prior negative biopsy status (yes/no), and PSA density (ng/ml/cc) based on transrectal ultrasound prostate volume.

### MRI scan and image interpretation

Pre-biopsy prostate MRI was conducted using a 1.5 Tesla AVANTO® MRI scanner (Siemens, Healthcare, Erlangen, Germany) or a 3 Tesla INGENIA® MRI scanner (Philips Medical Systems, Best, the Netherlands). MRI series consisted of at least T1-weighted, T2-weighted, diffusion-weighted imaging and calculation of apparent diffusion coefficient maps. All prostate MRIs were evaluated by dedicated uroradiologists (>5 years’ experience) according to the PI-RADS classification version 2 or 2.1.^11,12^

### Biopsy procedure

Systematic prostate biopsy (SBx) procedures were performed transrectally or transperineally by dedicated operators (>150 procedures/year), based on a modified Barzell template of the peripheral and transitional zone.^13^ Biopsy cores were retrieved using an 18G biopsy gun, independently labeled and fixated in separate containers for histological assessment of each pre-defined location within the prostate. This was evaluated by a dedicated uropathologist and graded according to the International Society of Urological Pathology (ISUP) consensus on grading of PCa.^14^

### Statistical analysis

Statistical analysis was performed using IBM SPSS Statistics (version 26). Demographic data were presented of the overall cohort and divided in subgroups based on pathology results: benign, ISUP 1, ISUP ⩾ 2, and ISUP ⩾ 3. Multivariable logistic regression analysis with backward elimination was performed to predict csPCa presence using relevant clinical parameters, using a cut-out value of *p* > 0.157.^15,16^ Odds ratios (ORs) and 95% confidence intervals (95% CIs) were presented for significant predictors. Receiver operating characteristic (ROC) curve analysis, based on significant predictors, was performed to determine area under the curve (AUC) values including 95% CI for the Prostate Biopsy Cohort Amsterdam (‘PROBA’) RC. A Delong test was used to compare the AUC values of the ‘PROBA RC’ with the calculated risk results of the same population using the RCs of the European Randomized Study of Screening for Prostate Cancer (ERSPC), Prostate Biopsy Collaborative Group (PBCG), and Prospective Loyola University mpMRI (PLUM). Clinical utility of the ‘PROBA RC’ was assessed based on negative predictive values (NPV) of the optimal risk threshold, calculated by the Youden index, and compared with the other RCs.

## Results

**Figure 1.**
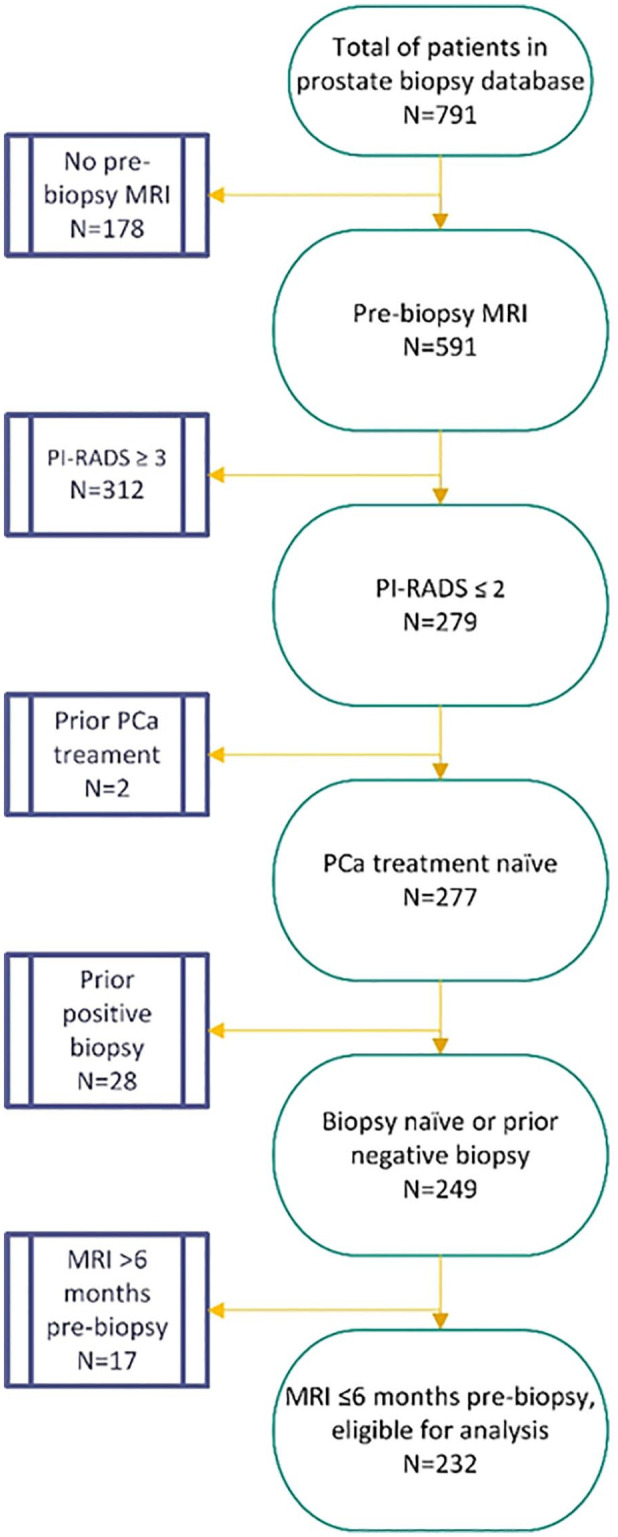
Flowchart of patients included for analysis. MRI, magnetic resonance imaging; PCa, prostate cancer; PI-RADS, Prostate Imaging–Reporting and Data System.

The database consisted of a total of 790 men, of which 232 men were eligible for analysis (Figure 1). This cohort consisted of 188 biopsy-naïve (81%) and 44 prior negative biopsy men (19%). The overall median (interquartile range (IQR)) age was 64 (10) years. The median (IQR) pre-biopsy PSA was 6.5 (4.1) ng/ml and median (IQR) prostate volume of 55 (34) cc. This resulted in a median (IQR) PSA density of 0.12 (0.09) ng/ml/cc. A suspicious digital rectal exam was identified in 40 men (17.2%) and a family history of PCa was found in 29 men (12.5%). Table 1 provides an overview of all patient characteristics.

**Table 1.** Characteristics of patients with negative (PI-RADS 1-2) MRI and who subsequently underwent systematic prostate biopsy.

		Prostate biopsy outcome			
	Overall	Benign	ISUP 1	ISUP ⩾ 2	ISUP ⩾ 3
No. of patients, *n* (%)	232 (100)	147 (100)	45 (100)	40 (100)	11 (100)
Median age (IQR)	64 (10)	64 (9)	65 (13)	65 (13.8)	65 (18)
Ancestry					
African Caribbean	49 (21.1)	29 (19.7)	5 (11.1)	15 (37.5)	4 (36.4)
Other	183 (78.9)	118 (80.3)	40 (88.9)	25 (62.5)	7 (63.6)
Suspicious digital rectal exam, *n* (%)	40 (17.2)	23 (15.6)	8 (17.8)	9 (22.5)	3 (27.3)
Positive family history of PCa, *n* (%)	29 (12.5)	13 (8.8)	3 (6.7)	13 (32.5)	5 (45.5)
Median PSA (IQR), ng/ml	6.5 (4.1)	6.3 (4)	6.1 (4.8)	7.4 (4.1)	7.6 (3.9)
Median prostate volume (IQR), ml	55 (34)	61.3 (36)	46 (25)	44.9 (29)	43 (32.8)
Median PSA density (IQR)	.12 (.09)	.12 (.09)	.13 (.07)	.16 (.14)	.18 (.14)
Biopsy status, *n* (%)					
Biopsy naïve	188 (81)	115 (78.2)	38 (84.4)	35 (87.5)	7 (63.6)
Prior negative biopsy	44 (19)	32 (21.8)	7 (15.5)	5 (12.5)	4 (36.4)
Median (IQR) ERSPC RC result	2 (2)	2 (2)	2 (3)	2 (2.8)	3 (3)
Median (IQR) PBCG RC result	29 (21)	27 (17)	29 (24)	35 (31)	38 (19)
Median (IQR) PLUM RC result	7.2 (9.8)	5.3 (8.6)	8.6 (6.9)	13 (16.6)	7.8 (17.4)
MRI field strength					
1.5 Tesla	92 (39.7)	53 (36.1)	23 (51.1)	17 (42.5)	5 (45.5)
3.0 Tesla	140 (60.3)	94 (63.9)	22 (48.9)	23 (57.5)	6 (54.5)
Biopsy approach, *n* (%)					
Transrectal	198 (85.3)	130 (88.4)	35 (77.8)	34 (85)	9 (81.8)
Transperineal	34 (14.7)	17 (11.6)	10 (22.2)	6 (15)	2 (18.2)
Median amount of biopsy cores (IQR)	13 (3)	12 (2)	12 (2)	12 (1.8)	15 (3)

ERSPC, European Randomized Study of Screening for Prostate Cancer; IQR, interquartile range; ISUP, International Society of Urological Pathology; MRI, magnetic resonance imaging; PBCG, Prostate Biopsy Collaborative Group; PI-RADS, Prostate Imaging–Reporting and Data System; PLUM, Prospective Loyola University mpMRI; PSA, prostate-specific antigen; RC, risk calculator.

### Performance of ‘PROBA RC’ compared with other RC

**Figure 2.**
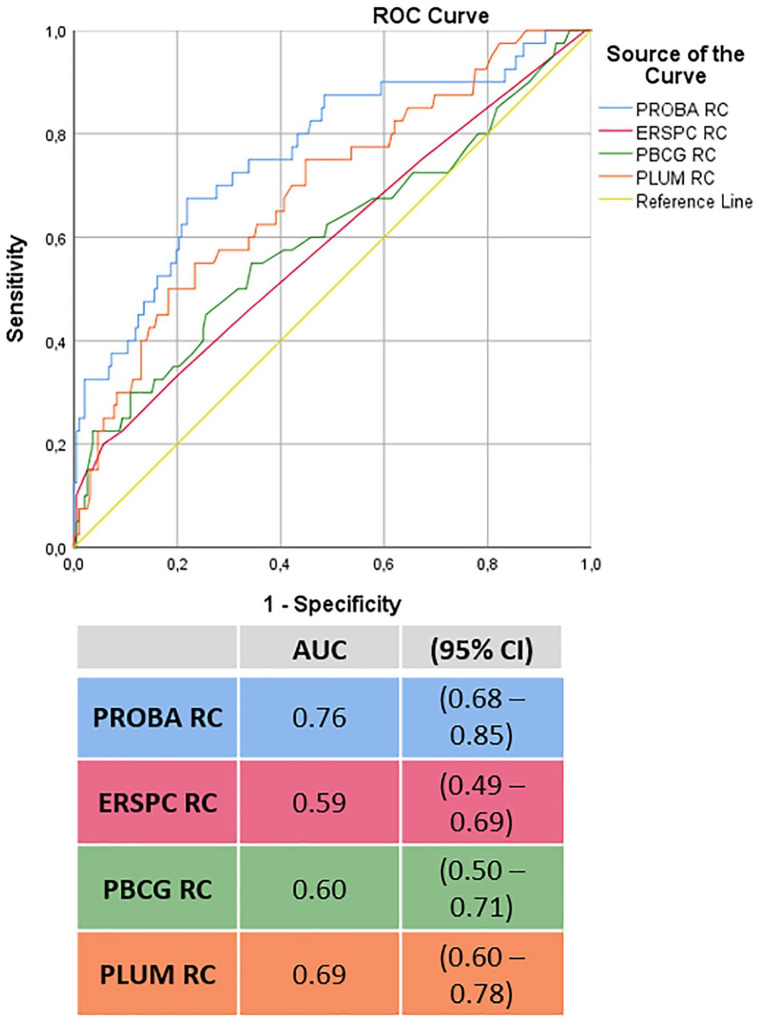
Receiver operating characteristic (ROC) curve analysis of the Prostate Biopsy Cohort Amsterdam (PROBA; blue line) risk calculator compared with the European Randomized Study of Screening for Prostate Cancer (ERSPC; pink line), Prostate Biopsy Collaborative Group (PBCG; green line), and Prospective Loyola University mpMRI (PLUM; orange line) risk calculators for presence of International Society of Urological Pathology grade group ⩾ 2 upon systematic prostate biopsy.

Systematic prostate biopsy detection rates of PCa and csPCa were 36.6% (85 men) and 17.2% (40 men), respectively. ISUP ⩾ 3 was detected in 11 men (4.7%). Multivariable logistic regression analysis showed that a family history of PCa (*p* < 0.0001), African Caribbean ancestry (*p* = 0.005), PSA density (*p* = 0.002), prior negative biopsy (*p* = 0.063), and age at biopsy (*p* = 0.157) were associated with csPCa presence (Table 2), and, therefore, included in the final ‘PROBA RC’. The AUC of the ‘PROBA RC’ 0.76 (95% CI 0.68 – 0.85) was higher than the AUC of the RCs of the ERSPC (*p* = 0.001), PBCG (*p* = 0.002) and PLUM (*p* = 0.09), which were 0.59 (95% CI 0.49 – 0.69), 0.60 (95% CI 0.50 – 0.71), and 0.69 (95% CI 0.60 – 0.78), respectively, see Figure 2.

**Table 2.** Predictors for ISUP ⩾ 2 in systematic prostate biopsy pathology result in patients with a negative-MRI.

Clinical parameters	Multivariable logistic regression		
	OR	(95% CI)	*p* value
Age at biopsy	1.04	(0.99–1.09)	0.157
Positive family history of PCa	8.42	(3.15–22.56)	<0.0001
African Caribbean ethnicity	3.34	(1.44–7.78)	0.005
PSA density	3.21	(1.56–6.63)	0.002
Prior negative biopsy	0.34	(0.11–1.06)	0.063

95% CI, 95% confidence interval; ISUP, International Society of Urological Pathology grade group; MRI, magnetic resonance imaging; OR, odds ratio; PCa, prostate cancer; PSA, prostate-specific antigen.

The NPV of negative-MRI in the overall cohort for absence of csPCa was 82.8% (192/232). The optimal threshold-probability of the ‘PROBA RC’ for prediction of csPCa presence was determined at 20%. See Supplementary Material for an overview of NPV at differing threshold-probabilities. When applying the ‘PROBA RC’ at the optimal threshold, the NPV of negative-MRI increased to 92% (150/163) for ISUP ⩾ 2 and to 98.2% (160/163) for ISUP ⩾ 3 in the overall cohort. The ‘PROBA RC’ outperformed other RCs regarding NPV in the overall cohort, and in biopsy subgroups (Table 3).

**Table 3.** Negative predictive values for ISUP ⩾ 2 and ISUP ⩾ 3 absence in patients with a negative-MRI at prostate biopsy and different RCs at their optimal threshold-probability.

Risk threshold	ISUP ⩾ 2			ISUP ⩾ 3		
	Overall, *n* (%)	Biopsy naïve, *n* (%)	Prior negative biopsy, *n* (%)	Overall, *n* (%)	Biopsy naïve, *n* (%)	Prior negative biopsy, *n* (%)
MRI alone	192/232 (82.8)	153/188 (81.4)	39/44 (88.6)	221/232 (95.3)	181/188 (96.3)	40/44 (90.9)
PROBA RC < 20%	150/163 (92)	117/128 (91.4)	33/35 (94.3)	160/163 (98.2)	127/128 (99.2)	33/35 (94.3)
RC of ERSPC < 5.5%	181/213 (85)	142/169 (84)	39/44 (88.6)	204/213 (95.8)	164/169 (97)	40/44 (90.9)
RC of PBCG < 33.5%	126/144 (87.5)	95/108 (88)	31/36 (86.1)	139/144 (96.5)	107/108 (99.1)	32/36 (88.9)
RC of PLUM < 14%	157/177 (88.7)	120/137 (87.6)	37/40 (92.5)	171/177 (96.6)	134/137 (97.8)	37/40 (92.5)

ERSPC, European Randomized Study of Screening for Prostate Cancer; ISUP, International Society of Urological Pathology grade group; MRI, magnetic resonance imaging; PBCG, Prostate Biopsy Collaborative Group; PLUM, Prospective Loyola University mpMRI; PROBA, Prostate Biopsy Cohort Amsterdam; RC, risk calculator.

Based on a 20% risk threshold for the ‘PROBA RC’, a total of 70.2% (163/232) of SBx could have been avoided, at the cost of missing 5.6% (13/232) csPCa cases, while decreasing iPCa detection by 15.9% (37/232). Based on the ERSPC RC, at the optimal threshold of 5.5%, 91.8% (213/232) of SBx could have been avoided, leading to 13.8% (32/232) missed csPCa cases and preventing detection of 17.7% (41/232) iPCa. Using the PBCG RC, at the optimal threshold of 33.5%, would have resulted in a 62.1% (144/232) reduction of SBx, 7.7% (18/232) missed csPCa, and a 12.1% (28/232) decrease in iPCa detection. Finally, the PLUM RC, at the optimal threshold of 14%, could have avoided 76.3% (177/232) of SBx, at the cost of 8.6% (20/232) missed csPCa, while reducing iPCa detection by 15.5% (36/232).

### Details on men with csPCa on SBx missed by the ‘PROBA RC’

When applying a risk threshold of 20% for the ‘PROBA RC’, a total of 13 csPCa men would have been missed. Out of these 13 men with csPCa at SBx, histopathology showed ISUP 2 in 10 men and ISUP 3 in the remaining 3. Cribriform growth was not observed in any of the positive cores. The median (IQR) number of positive cores was 1 (1) for both ISUP 2 and 3 men, with a median (IQR) maximum cancer core length (MCCL) of 4.3 (1.8) mm. The median (IQR) number of total SBx cores was 12 (2).

## Discussion

A negative-MRI alone was found to be inadequate to omit SBx, with an NPV of 82.8% for csPCa. Based on identification of independent predictors for csPCa in men with a pre-biopsy negative-MRI, we constructed an RC, the PROBA RC.

The risk factors were family history for PCa, African Caribbean ancestry, PSA density, prior negative biopsy status, and age at biopsy. This ‘PROBA RC’ showed to be of significant added value for selecting men, in whom prostate biopsies could be omitted following negative-MRI. The ‘PROBA RC’ increased the NPV of prostate MRI to 92% and reached an AUC of 0.76 (95% CI 0.68–0.85), thereby outperforming the ERSPC, PBCG, and PLUM RCs, with AUCs of 0.59, 0.60, and 0.69, respectively.

When comparing the different RCs, notable dissimilarities can be found in the predictive factors that were included. The ERSPC RC includes age, PSA, DRE, prostate volume, prior negative biopsy, and MRI results. In a contemporary Dutch cohort of 2270 prostate biopsy men, this RC achieved a similar AUC as the ‘PROBA RC’ in the current study (0.76). However, MRI results were not included in this study.^8^ The PBCG RC includes ancestry, age, PSA, DRE, biopsy status, and family history of PCa. Yet, MRI results are not implemented in the PBCG RC. External validation on several large cohorts showed an AUC of 0.76.^17^ The PLUM RC includes age, PSA, DRE, prostate volume, ancestry, family history of PCa, prior negative biopsy, and MRI results, which resulted in an AUC of 0.88 for biopsy naïve and 0.87 for prior negative biopsy men. This seems a promising RC, yet it has not been externally validated.

Where the ‘PROBA’, PBCG, and PLUM RCs include ancestry and family history, the ERSPC RC does not. The role of ancestry for the prediction of csPCa remains controversial, yet it is suggested by the European guidelines as a potential risk factor.^5^ In a recent meta-analysis on predictive factors for csPCa in negative-MRI men, ancestry was investigated in one study, and it was not found to be a significant predictor.^9^ However, epidemiological studies have shown a higher prevalence of PCa in populations of African descent (OR 3.34).^18^ In the present study, an association between African Caribbean ancestry and csPCa was confirmed (*p* = 0.005).

Family history has also shown to be related to csPCa with incidence ratios of up to 4.0 for first degree relatives in population-based studies.^19–21^ In our study, family history for PCa was the predictor with the largest association with csPCa, with an OR of 8.42. The fact that this factor is not included in the ERSPC RC might explain the underperformance compared with the ‘PROBA’ and PLUM RCs. The PBCG RC still performed similarly to the ERSPC RC, possibly because it does not include MRI results.

In all investigated RCs, PSA and prostate volume are included. In the current cohort, a higher PSA density was significantly related to the presence of csPCa. In the recent meta-analysis by Pagniez *et al.*,^9^ PSA density was the most relevant predictive factor of csPCa presence in biopsy-naïve and prior negative biopsy men. They showed that the NPV of a negative-MRI for csPCa presence improved, when selecting a PSA density cut-off of  < 0.15 ng/ml/cc, in both biopsy-naïve (82.7–88.7%) and prior negative biopsy men (88.2–94.1%).

Furthermore, in this meta-analysis, two studies were included assessing the predictive value of a prior negative biopsy status. Both studies found that a prior negative biopsy status was significantly associated with the absence of csPCa, with an OR up to 5.2 (95% CI 1.6–16.5; *p* = 0.005). In the present study, similar results were found regarding the prior negative biopsy status.

To our knowledge, this is the first study assessing predictors for the presence of csPCa in men with a negative-MRI. Whether to implement a novel RC in regular practice depends mainly on the clinical implications of applying the RC. By only performing SBx in men with an ‘PROBA RC’: ⩾ 20%, 163 out of 232 (70.3%) men could have prevented SBx, leading to a reduction of 37 (15.9%) iPCa cases detected at the cost of 13 (5.6%) missed csPCa cases. Interestingly, none of the missed csPCa cases showed high-risk characteristics, all being ISUP ⩽ 3, without cribriform growth and a low number of positive biopsy cores. Five out of these 13 (38.5%) men with missed csPCa did not receive active treatment, considering they were eligible for active surveillance.^22^ Therefore, arguably, only 7 clinically relevant PCa cases out of 232 (3%) SBx patients were missed. We conclude that SBx can be safely omitted in selected men with a negative-MRI and a risk of  < 20% for the ‘PROBA RC’.

This study has several limitations. Although the ‘PROBA RC’ showed promising results for men with a negative-MRI, it requires external validation to evaluate its applicability in general practice. Besides, diagnostic accuracy of both MRI and prostate biopsy is dependent on locally available expertise of radiologists and biopsy operators.^23–25^ Consequently, the applicability of RCs will differ depending on the clinic. The discrepancy between the predictive values of our local ‘PROBA RC’ and other RCs highlights this issue and shows the importance of locally evaluating MRI and biopsy performance. Also, due to its retrospective design, this study is at risk for selection bias. Moreover, it is limited by a relatively small sample size. In addition, the reference standard was SBx and not template biopsies, which might underestimate the presence of csPCa. Furthermore, performance of the ‘PROBA RC’ could be further increased by incorporating other risk factors, for example, family history of BRCA2 mutation, hereditary breast and ovarian cancer, and Lynch syndrome.^26,27^

Whether to perform SBx in men with a negative-MRI remains a topic of discussion. The long-term clinical implication of omitting SBx is an important factor in this discussion. No high-level, prospective evidence is available to support omitting SBx in all men with a negative-MRI. Currently, the decision to proceed with SBx after a negative-MRI should be based on patient-specific characteristics. The use of RCs can aid in clinical decision making, but the accuracy of these calculators might vary between centers. Local evaluation of biopsy and prostate MRI results is paramount to guarantee the best quality of care for each patient.

## Conclusion

Shared decision making for performing SBx in men with a negative prostate MRI can be improved based on family history of PCa, African Caribbean ancestry, PSA density, prior negative biopsy status, and age at biopsy. The ‘PROBA RC’, developed in the present study integrating these predictors, performed superior when compared with widely available RCs. These findings require external validation before introduction in daily practice.

## Supplemental Material

sj-docx-1-tau-10.1177_17562872221088536 – Supplemental material for Predictors of clinically significant prostate cancer in biopsy-naïve and prior negative biopsy men with a negative prostate MRI: improving MRI-based screening with a novel risk calculator Click here for additional data file. Supplemental material, sj-docx-1-tau-10.1177_17562872221088536 for Predictors of clinically significant prostate cancer in biopsy-naïve and prior negative biopsy men with a negative prostate MRI: improving MRI-based screening with a novel risk calculator by Luigi A.M.J.G. van Riel, Auke Jager, Dennie Meijer, Arnoud W. Postema, Ruth S. Smit, André N. Vis, Theo M. de Reijke, Harrie P. Beerlage and Jorg R. Oddens in Therapeutic Advances in Urology

## References

[1] Ahmed HU El-Shater Bosaily A Brown LC , et al. Diagnostic accuracy of multi-parametric MRI and TRUS biopsy in prostate cancer (PROMIS): a paired validating confirmatory study. Lancet 2017; 389: 815–822. 28110982 10.1016/S0140-6736(16)32401-1

[2] Kasivisvanathan V Rannikko AS Borghi M , et al. MRI-targeted or standard biopsy for prostate-cancer diagnosis. N Engl J Med 2018; 378: 1767–1777. 29552975 10.1056/NEJMoa1801993 PMC9084630

[3] Porpiglia F Manfredi M Mele F , et al. Diagnostic pathway with multiparametric magnetic resonance imaging versus standard pathway: results from a randomized prospective study in biopsy-naive patients with suspected prostate cancer. Eur Urol 2017; 72: 282–288. 27574821 10.1016/j.eururo.2016.08.041

[4] Panebianco V Barchetti F Sciarra A , et al. Multiparametric magnetic resonance imaging vs. standard care in men being evaluated for prostate cancer: a randomized study. Urol Oncol 2015; 33: 17.e1–17.e7. 10.1016/j.urolonc.2014.09.013 25443268

[5] Mottet N van den Bergh RCN Briers E , et al. EAU-EANM-ESTRO-ESUR-SIOG guidelines on prostate cancer-2020 update. Part 1: screening, diagnosis, and local treatment with curative intent. Eur Urol 2021; 79: 243–262. 33172724 10.1016/j.eururo.2020.09.042

[6] Drost FH Osses D Nieboer D , et al. Prostate magnetic resonance imaging, with or without magnetic resonance imaging-targeted biopsy, and systematic biopsy for detecting prostate cancer: a Cochrane systematic review and meta-analysis. Eur Urol 2020; 77: 78–94. 31326219 10.1016/j.eururo.2019.06.023

[7] Louie KS Seigneurin A Cathcart P , et al. Do prostate cancer risk models improve the predictive accuracy of PSA screening? A meta-analysis. Ann Oncol 2015; 26: 848–864. 25403590 10.1093/annonc/mdu525

[8] Gayet M Mannaerts CK Nieboer D , et al. Prediction of prostate cancer: external validation of the ERSPC risk calculator in a contemporary Dutch clinical cohort. Eur Urol Focus 2018; 4: 228–234. 28753781 10.1016/j.euf.2016.07.007

[9] Pagniez MA Kasivisvanathan V Puech P , et al. Predictive factors of missed clinically significant prostate cancers in men with negative magnetic resonance imaging: a systematic review and meta-analysis. J Urol 2020; 204: 24–32. 31967522 10.1097/JU.0000000000000757

[10] US Preventive Services Task Force, Grossman DC Curry SJ , et al. Screening for prostate cancer: US Preventive Services Task Force recommendation statement. JAMA 2018; 319: 1901–1913. 29801017 10.1001/jama.2018.3710

[11] Turkbey B Rosenkrantz AB Haider MA , et al. Prostate Imaging Reporting and Data System version 2.1: 2019 update of Prostate Imaging Reporting and Data System version 2. Eur Urol 2019; 76: 340–351. 30898406 10.1016/j.eururo.2019.02.033

[12] Weinreb JC Barentsz JO Choyke PL , et al. PI-RADS Prostate Imaging – Reporting and Data System: 2015, version 2. Eur Urol 2016; 69: 16–40. 26427566 10.1016/j.eururo.2015.08.052 PMC6467207

[13] Barzell WE Melamed MR . Appropriate patient selection in the focal treatment of prostate cancer: the role of transperineal 3-dimensional pathologic mapping of the prostate – a 4-year experience. Urology 2007; 70(Suppl. 6): 27–35. 18194708 10.1016/j.urology.2007.06.1126

[14] Egevad L Delahunt B Srigley JR , et al. International Society of Urological Pathology (ISUP) grading of prostate cancer – an ISUP consensus on contemporary grading. APMIS 2016; 124: 433–435. 27150257 10.1111/apm.12533

[15] Moons KG Altman DG Reitsma JB , et al. Transparent Reporting of a multivariable prediction model for Individual Prognosis or Diagnosis (TRIPOD): explanation and elaboration. Ann Intern Med 2015; 162: W1–W73. 25560730 10.7326/M14-0698

[16] Steyerberg EW . Clinical prediction models: a practical approach to development, validation, and updating. New York: Springer, 2009.

[17] Ankerst DP Straubinger J Selig K , et al. A contemporary prostate biopsy risk calculator based on multiple heterogeneous cohorts. Eur Urol 2018; 74: 197–203. 29778349 10.1016/j.eururo.2018.05.003 PMC6082177

[18] Ferlay J Soerjomataram I Dikshit R , et al. Cancer incidence and mortality worldwide: sources, methods and major patterns in GLOBOCAN 2012. Int J Cancer 2015; 136: E359–E386. 10.1002/ijc.29210 25220842

[19] Jansson KF Akre O Garmo H , et al. Concordance of tumor differentiation among brothers with prostate cancer. Eur Urol 2012; 62: 656–661. 22386193 10.1016/j.eururo.2012.02.032

[20] Jansson F Drevin L Frisell T , et al. Concordance of non-low-risk disease among pairs of brothers with prostate cancer. J Clin Oncol 2018; 36: 1847–1852. 29652556 10.1200/JCO.2017.76.6907

[21] Hemminki K . Familial risk and familial survival in prostate cancer. World J Urol 2012; 30: 143–148. 22116601 10.1007/s00345-011-0801-1

[22] Guideline and study for the expectant management of localized prostate cancer with curative intent: Prostate Cancer Research International: Active Surveillance (PRIAS), 2020, http://prias-project.org

[23] Brembilla G Dell’Oglio P Stabile A , et al. Interreader variability in prostate MRI reporting using Prostate Imaging Reporting and Data System version 2.1. Eur Radiol 2020; 30: 3383–3392. 32052171 10.1007/s00330-019-06654-2

[24] Kohestani K Wallstrom J Dehlfors N , et al. Performance and inter-observer variability of prostate MRI (PI-RADS version 2) outside high-volume centres. Scand J Urol 2019; 53: 304–311. 31661357 10.1080/21681805.2019.1675757 PMC6935323

[25] Tadtayev S Hussein A Carpenter L , et al. The association of level of practical experience in transrectal ultrasonography guided prostate biopsy with its diagnostic outcome. Ann R Coll Surg Engl 2017; 99: 218–223. 27659356 10.1308/rcsann.2016.0308 PMC5450273

[26] Beebe-Dimmer JL Kapron AL Fraser AM , et al. Risk of prostate cancer associated with familial and hereditary cancer syndromes. J Clin Oncol 2020; 38: 1807–1813. 32208047 10.1200/JCO.19.02808 PMC7255976

[27] Oh M Alkhushaym N Fallatah S , et al. The association of BRCA1 and BRCA2 mutations with prostate cancer risk, frequency, and mortality: a meta-analysis. Prostate 2019; 79: 880–895. 30900310 10.1002/pros.23795

