# Supplementary material for: Predictors of clinically significant prostate cancer in biopsy-naïve and prior negative biopsy men with a negative prostate MRI: improving MRI-based screening with a novel risk calculator
Source: Ther Adv Urol. 2022 Mar 26;14:17562872221088536. doi: 10.1177/17562872221088536 (PMC8958520; doi:10.1177/17562872221088536)
Supplement: sj-docx-1-tau-10.1177_17562872221088536 – Supplemental material for Predictors of clinically significant prostate cancer in biopsy-naïve and prior negative biopsy men with a negative prostate MRI: improving MRI-based screening with a novel risk calculator [file sj-docx-1-tau-10.1177_17562872221088536.docx]

| **Supplementary material: Negative predictive values for ISUP 1, ISUP ≥2 and ISUP ≥3 absence in biopsy naïve and prior negative biopsy patients with a negative MRI at prostate biopsy based on the AUMC RC and different threshold-probabilities values.** | | | | | | | | | |  |
| --- | --- | --- | --- | --- | --- | --- | --- | --- | --- | --- |
|  | **Overall** | | | **Biopsy naïve** | | | **Prior negative biopsy** | | | **Youden index** |
| **RC and risk threshold** | **ISUP 1** | **ISUP ≥2** | **ISUP ≥3** | **ISUP 1** | **ISUP ≥2** | **ISUP ≥3** | **ISUP 1** | **ISUP ≥2** | **ISUP ≥3** |  |
| AUMC RC <10% | 79.8 (79/99) | 94.9 (94/99) | 98 (97/99) | 79.7 (55/69) | 95.7 (66/69) | 100 (69/69) | 80 (24/30) | 93.3 (28/30) | 93.3 (28/30) | 0.37 |
| AUMC RC <12% | 79.2 (95/120) | 92.5 (111/120) | 98.3 (118/120) | 78.2 (68/87) | 92 (80/87) | 100 (87/87) | 81.8 (27/33) | 93.9 (31/33) | 93.9 (31/33) | 0.35 |
| AUMC RC <14% | 79.7 (102/128) | 92.2 (118/128) | 98.4 (126/128) | 79 (75/95) | 91.6 (87/95) | 100 (95/95) | 81.8 (27/33) | 93.9 (31/33) | 93.9 (31/33) | 0.37 |
| AUMC RC<16% | 79.3 (111/140) | 92.1 (129/140) | 97.9 (137/140) | 78.5 (84/107) | 91.6 (98/107) | 99.1 (106/107) | 81.8 (27/33) | 93.9 (31/33) | 93.9 (31/33) | 0.40 |
| AUMC RC <18% | 77.9 (120/154) | 91.6 (141/154) | 98.1 (151/154) | 76.5 (91/119) | 90.8 (108/119) | 99.2 (118/119) | 82.9 (29/35) | 94.3 (33/35) | 94.3 (33/35) | 0.40 |
| AUMC RC <20% | 77.3 (126/163) | 92 (150/163) | 98.2 (160/163) | 75.8 (97/128) | 91.4 (117/128) | 99.2 (127/128) | 82.9 (29/35) | 94.3 (33/35) | 94.3 (33/35) | 0.46 |
| AUMC RC <22% | 78.5 (135/172) | 89.5 (154/172) | 97.7 (168/172) | 77.4 (106/137) | 88.3 (121/137) | 98.5 (135/137) | 82.9 (29/35) | 94.3 (33/35) | 94.3 (33/35) | 0.35 |
| AUMC RC <24% | 78.9 (142/180) | 89.4 (161/180) | 97.2 (175/180) | 77.9 (113/145) | 88.3 (128/145) | 97.9 (142/145) | 82.9 (29/35) | 94.3 (33/35) | 94.3 (33/35) | 0.36 |
| AUMC RC <26% | 79.5 (147/185) | 88.6 (164/185) | 97.3 (180/185) | 78.4 (116/148) | 87.8 (130/148) | 98 (145/148) | 83.8 (31/37) | 91.9 (34/37) | 94.6 (35/37) | 0.33 |
| AUMC RC <28% | 80.3 (155/193) | 87.6 (169/193) | 96.9 (187/193) | 79.2 (122/154) | 87 (134/154) | 98.1 (151/154) | 84.6 (33/39) | 89.7 (35/39) | 92.3 (36/39) | 0.31 |
| AUMC RC <30% | 80.6 (158/196) | 87.8 (172/196) | 96.9 (190/196) | 79. 5 (124/156) | 87.2 (136/156) | 98.1 (153/156) | 85 (34/40) | 90 (36/40) | 92.5 (37/40) | 0.30 |

**Abbreviations:** ISUP = International Society of Urological Pathology grade group, MRI = Magnetic Resonance Imaging and RC = Risk Calculator.
